# MYB4 in *Lilium pumilum* affects plant saline-alkaline tolerance

**DOI:** 10.1080/15592324.2024.2370724

**Published:** 2024-07-14

**Authors:** Fanru Zhang, Xiaochao Zhang, Wenhao Wan, Xingyu Zhu, Miaoxin Shi, Ling Zhang, Fengshan Yang, Shumei Jin

**Affiliations:** a Key Laboratory of Saline-alkali Vegetation Ecology Restoration, Ministry of Education, College of Life Sciences, Northeast Forestry University, Harbin, China; b Engineering Research Center of Agricultural Microbiology Technology, Ministry of Education & Heilongjiang Provincial Key Laboratory of Ecological Restoration and Resource Utilization for Cold Region & Key Laboratory of Molecular Biology, College of Heilongjiang Province & School of Life Sciences, Heilongjiang University, Harbin, China

**Keywords:** MYB4, *Lilium pumilum*, saline-alkaline stress, Thioredoxin, GPX6 - glutathione peroxidase 6

## Abstract

*Lilium pumilum* DC (*L. pumilum* DC) plays an important role in the rational utilization of salinized soil. To explore the molecular mechanism of salt-tolerant *L. pumilum*, the *LpMYB4* was cloned. LpMYB4 close relationship with *Bambusa emeiensis* and *Zea mays MYB4* throughout the phylogenetic tree construction. LpMYB4 protein was found to be localized in the nucleus. Prokaryotic and eukaryotic bacterial solution resistance experiments proved that the exogenous introduction of *LpMYB4* made the overexpression strains obtain better survival ability under saline-alkaline stress. Compared with wild-type plants, tobacco plants overexpressing *LpMYB4* had better growth and lower leaf wilting and lodging, the content of chlorophyll was higher, the content of hydrogen peroxide and superoxide anion was lower, the activity of peroxidase and superoxide dismutase was higher and the relative conductivity was lower under saline-alkaline stress. The analysis of seed germination and seedling resistance of transgenic plants under salt stress showed that *LpMYB4* transgenic seeds were more tolerant to salt stress during germination and growth. Yeast two-hybrid and two-luciferase complementation experiments showed that LpMYB4 interacted with yeast two-hybrid and LpGPX6. The analysis of the role of *LpMYB4* in improving plant saline-alkali resistance is helpful to the transformation of plant germplasm resources and has great significance for agriculture and sustainable development.

## Introduction

Due to worldwide industrialization, urbanization, and salinization, the land available for crop cultivation is rapidly decreasing, and more than 800 million hectares of cultivated land in the world have been affected by salinization.^1^ The main difference between plants and animals is that they cannot move autonomously, so they have to deal with various stresses in the soil (such as salt, alkali, drought, heavy metals, etc.) by themselves.^2^ China is a big country with saline-alkali land distribution, Songneng Plain in Northeast China is the largest soda-type saline-alkali land concentration area in China and the third largest in the world, accounting for more than 90% of the total saline-alkali land area in Northeast China, which seriously threatens agricultural and economic development.^3^ *Lilium pumilum* DC. (*L. pumilum* DC) is one of the few plants that can survive in salinized soil. It has good resistance to drought, salinization cold, and is an excellent germplasm resource. In addition, it also has important ornamental value, medicinal, edible and other economic value, and has the potential to treat various diseases, especially anti-inflammatory and antioxidant.^4^ 20 mM NaHCO_3_ solution treated *Lilium pumilum* bulbs for 24 h, transcriptome sequencing was performed using IlluminaHiSeqTM2000 platform for comparative analysis. Significant differences were found in the expression of 390 genes (He, 2020). The expression of *LpMYB4* was up-regulated. In this experiment, the role of *LpMYB4* gene in improving plant saline-alkali resistance was explored.

As one of the most widely distributed transcription factor families in plants, the first *MYB* transcription factor was first cloned from Gramineae in higher plants,^5^ *MYB* is involved in plant development and stress response by binding to cis-elements of target genes. The transcription factor MYB4 is R2R3-MYB, belonging to subgroup 4-MYB subprotein family.^6,7^ The *MYB4* can effectively regulate a variety of abiotic stress responses in plants, including saline-alkaline. *Arabidopsis thaliana* with overexpression of rice *OsMYB4* gene induced a significant increase in the expression of drought resistance gene *P5CS*. Overexpression of *OsMYB4* in apple plants resulted in a large accumulation of metabolites, which improved the ability of plants to resist drought stress.^8^ Physiological indexes of *Arabidopsis thaliana* derived from transgenic *PgMYB4* were measured, and it was found that *PgMYB4* gene could improve the drought-resistant ability of plants.^9^ Transgenic *BpMYB4* plants have higher proline and cellulose synthesis capacity and lower MDA content. In addition, they found that overexpression of *BpMYB4* can clear H_2_O_2_ and O_2_^−^ and show a lower degree of cell damage.^10^ The *MsMYB4* may be directly involved in alfalfa’s response to saline-alkaline stress.^11^ Overexpression of *PgMYB4* enhanced the saline-alkali tolerance of Arabidopsis seedling.^12^ *MsMYB4* is the main regulator of plant response to saline-alkaline stress, and saline-alkaline stress can induce epigenetic changes in alfalfa *MsMYB4* promotes.^6,11^ *MYB4* gene could be induced by saline-alkaline stress, and the transcription level reached a peak after 100 mM NaCl induction for 1 h and then decreased. The sensitivity of the mutant to NaCl was significantly increased, and the tolerance of overexpressed plants to saline-alkaline stress was significantly enhanced.^7^ As a key transcription factor in plants, MYB4 may activate the expression of these genes by binding to the promoters of target genes (HKT1, NHX4, SOS1, and SOS3) to improve the tolerance of plants to saline-alkali stress.^7^ Other studies have shown that *MYB4* gene is also related to the synthesis of lignin and anthocyanins, which may be related to the mechanism of *MYB4* improving plant abiotic stress tolerance.^13,14^

We conducted a biological analysis of *LpMYB4*, mRNA level expression analysis, Prokaryotic (*E. coli*), and eukaryotic (Yeast) bacterial solution resistance experiments. Overexpression plants and other studies to comprehensively understand how *LpMYB4* regulates and responds to molecular mechanisms under saline-alkali stress, and provide effective engineering strategies for improving plant saline-alkali tolerance.

## Materials and methods

### Plant materials and growing conditions

*L. pumilum* collected from Daqing City, Northeast China, where most of the soil is saline. Tobacco (Nicotiana benthamiana) obtained was previously stored in our laboratory. In addition, *L. pumilum* and tobacco were grown in a regulated growth chamber at 25 ± 2°C, 2000 Lux, 16 h of sunlight/8 h of darkness photoperiod, and 75–80% relative humidity.

### Cloning of LpMYB4 gene

The total RNA of *L. pumilum* was extracted using a plant RNA extraction kit (Omega Bio-Tek, USA), and cDNA of *L. pumilum* was obtained using one step RT-qPCR Kit (Cowin Biosciences, China). Through sequencing analysis, specific primers (*LpMYB4-F* and *LpMYB4-R*, as shown in Table S1) were designed to obtain the ORF (open reading frame) of *LpMYB4*, and sequence the amplified PCR products.

### LpMYB4 bioinformatics analysis

Upload the sequencing results of *LpMYB4* to the NCBI database (https://www.ncbi.nlm.nih.gov). The MYB sequences with high similarity were found out from NCBI data, and DNAMAN and MEGA7 were used to compare homologous sequences and make the evolutionary tree.

### *Expression of LpMYB4 in* L. pumilum

RNA from *L. pumilum* was extracted and reversed transcribed to obtain cDNA from the roots, bulbs, leaves, flowers, and seeds of *L. pumilum*. The qPCR primers (*LpMYB4 qPCR- F* and *LpMYB4 qPCR- R*) were designed based on the sequence of the *LpMYB4* (Table S1). The primes of *LpActin F* and *LpActin R* was used as a control (Table S1). A 20 μL reaction system (cDNA 1 μL, RT-qPCR *LpMYB4-F* 1 μL, RT-qPCR *LpMYB4-R* 1 μL, 2×SYBR Green mix 10ul, ddH_2_O 7 μL) was made with Ultra SYBR Mixture reagents (Cowin Biosciences, China). The reaction conditions were: 95°C per denaturation for 10 min; 95°C for 30 s, 55°C for 30 s, 72°C for 2 min in total 40 cycles. And RT-qPCR was performed using a real-time quantitative PCR instrument (Agilent Mx3000p, USA) according to the standard reaction condition designed procedure. Three replicates were performed for each group of samples.

### Inducible expression and purity of LpMYB4 protein

*LpMYB4* (*LpMYB4-Bam*H1-F and *LpMYB4-Xho*I-R) was cloned into pGEX-6p-3 to obtain a fusion protein expression vector carrying GST and LpMYB4, which was transformed into *E. coli* BL21 (DE3).

For small volume induction, 1.50 ml EP Tube was used and the bacterial solution at different induction times (1 h, 3 h and 5 h) were centrifuged at 13,000 rpm for 1 min, supernatants were removed and the bacterial precipitates were resuspended using 200 μl of PBS buffer (the samples can be stored at −20°C). The above samples were mixed with Loading Buffer, boiled water bath for 10 min and then left on ice for 5 min, SDS-PAGE electrophoresis (120 V for 30 mins, 160 V for 45 mins), protein gels were stained with the appropriate amount of protein stain for 1 h, decolored overnight and observed the following day.

Shake the bacteria, incubate the bacterial solution at 37°C to OD_600_ = 0.5, 1 mM IPTG, centrifuge for 5 mins to enrich the bacterial body after 0 h and 5 h of induction at 37°C, resuspend the bacterial body with 5 ml of 1×PBS, sonicate the bacterial solution until it is clarified (power 80W, frequency 30 Hz, 5 s ON, 5 s OFF, 30 mins), followed by centrifugation at 8000 rpm for 30 mins, retain the supernatant with the Precipitation, respectively, on the sample for SDS-PAGE (protein staining and decolorization and other experimental steps are consistent with the small amount of protein induction).

### Resistance analysis of LpMYB4 protein-expressing prokaryotic bacterial solution under saline and alkaline stresses

After overnight incubation (37°C 130 rpm), when the OD_600_ = 0.5, a final concentration of 1 mM IPTG was added, and the incubation was continued at 37°C, 200 rpm for 1 h. A series of saline and alkaline stress (0.8 mM NaCl, 0.1 M Na_2_CO_3_, 0.2 M NaHCO_3_) was added, and the solution without any treatment was used as a control. The OD values of the BL21 bacterial solution carrying the pGEX-6p-3 and pGEX-6p-3-*LpMYB4* solution were measured under OD_600_ conditions of UV spectrophotometer at different times (0, 1, 3, and 5 h) of induction in a shaker at 37°C at 130 rpm, respectively.

### Analysis of the response of yeast expressing LpMYB4 to salt alkali stress

The *LpMYB4*-T plasmid was amplified using the primers containing restriction sites (*LpMYB4*-BamHI-F and *LpMYB4*-XhoI-R), and ligation with pYES2 resulting in pYES2-LpMYB4. Transformation of pYES2-*LpMYB4* into competent yeast strain INVSC1. After induction of protein expression in transgenic yeast cells, the obtained broth (OD_600_  = 1). The induced yeast solution was sequentially diluted ten, one hundred, one thousand, ten thousand, and one hundred thousand times and 3 ml of the yeast solution was taken and inoculated into CK untreated, YPD solid medium supplemented with 1 M NaCl, 30 mM NaHCO_3_, 20 mM Na_2_CO_3_, and 3.4 mM H_2_O_2_. 3 μL of the yeast solution was inoculated into the above plate and incubated at 30°C for 2 d, and the results were observed and analyzed.

### Transformation and characterization of LpMYB4 overexpression tobacco

pCAMBIA2300-GFP-*LpMYB4* (*LpMYB4*-BamHI-F and *LpMYB4*-XbaI-R) was successfully constructed and transformed into Agrobacterium *EHA105* using electrical conversion method. The positive strains were transferred into the tobacco using the instantaneous injection method, subcellular localization analysis of LpMYB4 protein was performed.

Tobacco overexpressing the *LpMYB4* was obtained through infected with agrobacterium *EHA105* with pCAMBIA2300-GFP-*LpMYB4* using agrobacterium-mediated transformation; ^15^ plant genomic DNA was extracted using the Plant Genome Extraction Kit (Thermo Fisher Scientific, China), PCR identification of overexpressed tobacco using two primers (*LpMYB4*-BamHI-F and *LpMYB4*-XbaI-R).

### Resistance analysis of LpMYB4 overexpression tobacco under saline and alkaline stress

In order to investigate the effects of saline-alkaline stress on the germination of WT seeds and *LpMYB4* transgenic seeds, wild-type and *LpMYB4* transgenic T3 generation tobacco seeds were taken and cultured in 1/2 MS medium and 1/2 MS medium supplemented with 4 mM Na_2_CO_3_, 8 mM NaHCO_3_ and 125 mM NaCl, respectively, for 7 d. Each stress treatment (included control) had three replications.

In order to investigate the resistance analysis of transgenic plants seeding under saline-alkaline stress, two-week-old wild-type tobacco seedlings and T3 generation *LpMYB4* transgenic seedlings were arranged in 1/2 MS medium supplemented with 4 mM Na_2_CO_3,_ 8 mM NaHCO_3_ and 125 mM NaCl, the Petri plates were vertically positioned in order to visualize the root growth. Plants were photographed after stress treatment for 14 d.

T3 tobacco seeds successfully identified were collected, and the wild-type and overexpressed *LpMYB4* tobaccos with the same growth were taken and analyzed for saline-alkali tolerance.

The wild type and overexpressed tobacco with the same growth were taken and watered using 600 mM NaCl, 2 M Na_2_CO_3_, 500 mM NaHCO_3_, 2 M H_2_O_2_ solution to the tobacco planted in the soil, 250 mL per day, and the phenotypic changes were observed after 48 h and photographed and recorded. Using the chlorophyll meter SPAD-502Plus, the chlorophyll content of wild type and *LpMYB4* overexpressing tobacco was measured under a series of saline stresses (2 M H_2_O_2_, 600 mM NaCl, 2 M Na_2_CO_3_, 500 mM NaHCO_3_); the relative conductivity of tobacco leaves was determined using the standard immersion method.^15^ Hydrogen peroxide content in plants was calculated using a hydrogen peroxide assay kit (Gerace Biotechnology Co., Ltd, Suzhou). The content of superoxide anion was calculated using the method of Li Zhongguang et al. ^16^ The activities of POD and SOD in wild-type and overexpressed tobacco were determined according to the method of Caixia Sun.^17^

### Screening of LpMYB4 interacting proteins

*LpMYB4* was cloned into pGBKT7 vector (*LpMYB4-Eco*RI-F and *LpMYB4-Bam*HI-R were listed in Table S1), then transformed into Y2H Gold strain, and screened for proteins interacting with pGBKT7-*LpMYB4* from cDNA library. Yeast DNA was extracted and sequenced to obtain the interacting gene and protein sequences. Single clones that turned blue on SD-Trp-Leu-His-Ade+X-α-gal medium were inoculated into SD-Trp-Leu-His-Ade liquid medium containing triple-deficient amino acids, followed by PCR reactions according to the primers (T7-F and 3-AD-R in Table S1). For PCR products with only a single band, sequencing was performed. NCBI-Blast was used for homology comparison, and the relationship between each gene and the improvement of plant salinity resistance was analyzed concerning related literature.

### Luciferase complementation assay

pCAMBIA1300-cLUC-*LpMYB4* (*LpMYB4*-KpnI-F and *LpMYB4*-SalI-R), pCAMBIA1300-NLUC-*LpGPX6* (*LpGPX6*-KpnI-F and *LpGPX6*-SalI-R) and *LpTrx*-pCAMBIA1300-NLUC-*LpTrx*-(*LpTrx*-KpnI-F and *LpTrx*-SalI-R) and transformed into agrobacterium tumefaciens strain *EHA105*. Bacteria were suspended using buffer (10 mM MgCl_2_, 10 mM MES, 200 μM Acetosyringone) and injected into four-week-old N. Benthamian, incubated in the dark for 36 h, then injected with D-Luciferin potassium salt at the injection site, before being placed in the dark for 5 min. A Chemiluminescent Imaging System (Tanon-5200, Tanon, China) was used for photography.

### Yeast two-hybrid experiment

The successfully constructed pGBKT7-*LpMYB4* was transformed into the Y2HGold yeast strain. The screened interacting protein pGADT7-LpTrx or pGADT7-LpGPX6 was constructed into pGADT7 vector, and the identified recombinant plasmids pGBKT7-*LpMYB4* and PGADT7-LpTrx or pGADT7-LpGPX6 were co-transformed into Y2HGold yeast strain. It was cultured on SD/-Trp-Leu and SD/-Trp-Leu-His-Ade +X-α-gal + AbA solid medium.

## Result

### Cloning of the LpMYB4 gene

**Figure 1.**
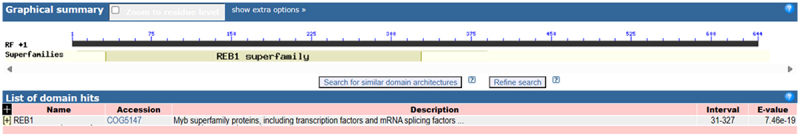
Analysis of the conserved domains of LpMYB4. Conservative domain analysis of LpMYB4 using NCBI. Result shows that there is a highly conserved MYB-DNA binding structural domain.

**Figure 2.**
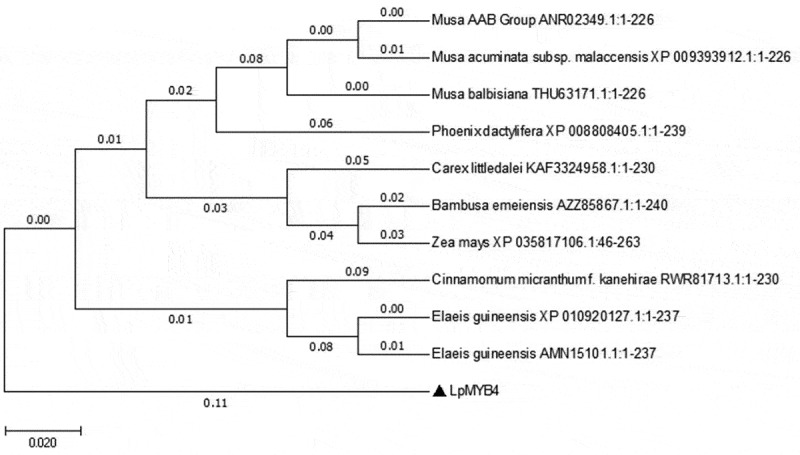
Phylogenetic tree analysis of LpMYB4. The scale bar represents that there is a 2% difference in this given length. The related species are as followsANR02349.1(ID): Musa AAB Group; XP 009393912.1: Musa acuminata subsp.Malaccensis;THU63171.1: Musa balbisiana; XP 008808405.1:Phoerix dactyifera; KAF3324958.1 Carex littledalei; AZZ85867.1: Bambusa emeiensis; XP035817106.1: Zea mays; RWR81713.1: Cinnamomum micranthumf kane hirae; XP010920127.1: Elaeis guineensis; AMN15101.1: Elaeis guineensis.

The *LpMYB4* gene was successfully cloned from the leaf of *Lilium pumilum*, the ORF of *LpMYB4* was 642bp, containing 213 amino acids, and according to the NCBI conserved structural domains analysis, LpMYB4 contained MYB-DNA binding structural domains, PLN03091, REB1, SANT, and other typical structural domains of MYB (Figure 1). The homologous sequence of *LpMYB4* was analyzed by NCBI Blast comparison and DNAMAN results (Fig. S1), and the amino acid highly conserved sequence is the black region and the similar sequence is the blue region, and it was found that the protein LpMYB4 has a high degree of homology with MYB proteins originating from other species (*American planthopper*, *small-fruited wild plantain*, *wild banana, karat banana*, *jujube, Dioscorea opposita*, *Konzang tarragon*, *Cichlidium*, and *Zeamays*). The related sequences were input into MEGA11 for evolutionary tree-building analysis, which revealed that LpMYB4 was more closely related to MYB proteins from Cichorium and maize (Figure 2), and these proteins may have similar functions.

### Analysis of LpMYB4 mRNA expression level

**Figure 3.**
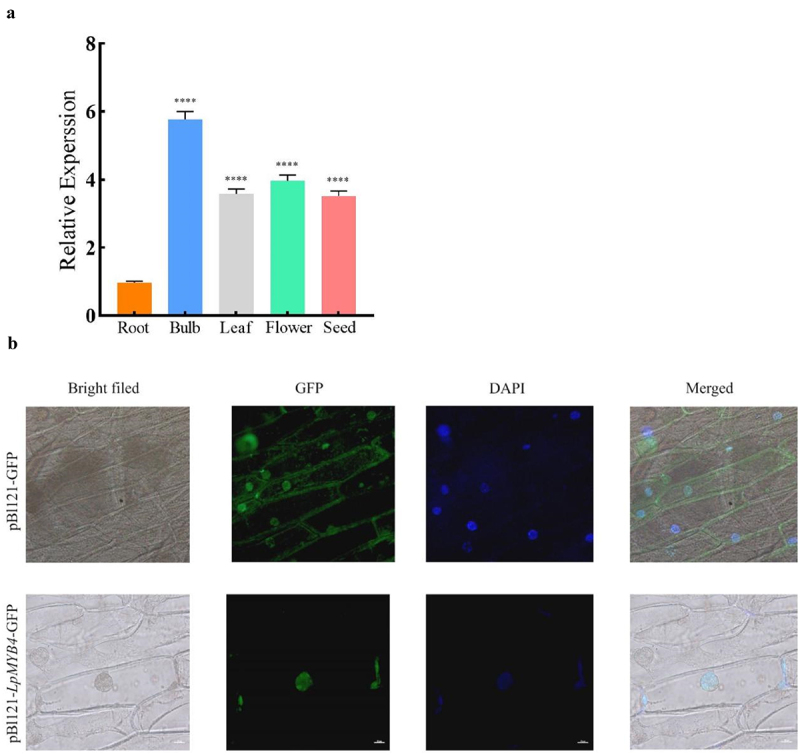
(a) RT-PCR analysis of the expression levels of LpMYB4 genes in different organs in *Lilium pumilum*. cDNA was obtained from the roots, bulbs, leaves, flowers, and seeds of *Lilium pumilum*, and the expression levels of LpMYB4 were detected by Real-time Quantitative PCR. ****respectively indicate that there is an extremely significant difference at *p* < 0.0001. Data represent mean ± SD with three replicates. (b) Localization of LpMYB4 in subcellular cells of onion.

The cDNAs of roots, bulbs, leaves, flowers and seeds in *L. Pumilum* were used as templates for RT-qPCR. The results showed that the *LpMYB4* was expressed in all organs tested in *L. Pumilum*, except for a relatively low expression in the root organ (Figure 3a).

### Subcellular localization of LpMYB4 protein

The pCAMBIA2300-GFP protein was discovered in the whole cell in transformed onion, The pCAMBIA2300-GFP-LpMYB4 protein was discovered in the nucleus (Figure 3b), which indicates that the LpMYB4 protein is mainly localized in the nucleus of the plant cell and that LpMYB4 serves as a transcription factor, and this result fits the expectation.

### Inducible expression of LpMYB4 protein

**Figure 4.**
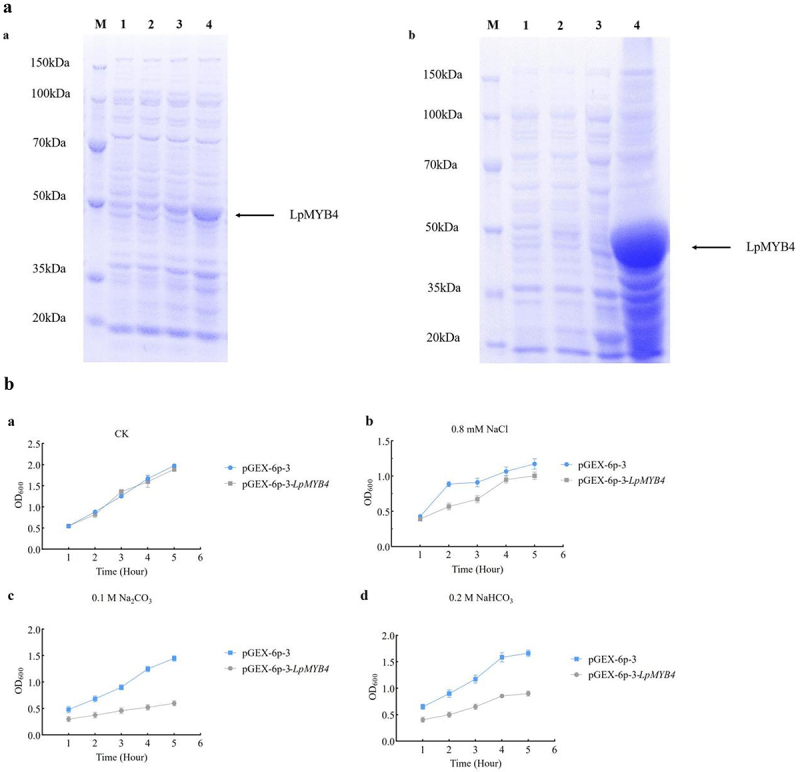
(a) Optimization of protein induction conditions and mass induction. a: Small volume induction of protein (M: protein marker; 1, 2, 3, 4, the expression of the protein was induced by 0, 1, 3, 5 h) b: massive protein induction; 1: 0 h of induction; 2: 5 h of induction; 3: supernatant; 4: sediment. (B) E. coli bacterial solution resistance analysis. a: The OD_600_ value of pGEX-6p-3 and pGEX-6p-3-LpMYB4 induced for 5 h under untreated conditions. (CK). b: The OD_600_ value of bacterial solution after 5 h induction by pGEX-6p-3 and pGEX-6p-3-LpMYB4 under the treatment of 0.8 mM NaCl. c: The OD_600_ value of bacterial liquid after 5 h induction by pGEX-6p-3 and pGEX-6p-3-LpMYB4 under the treatment of 0.1 mM Na_2_CO_3_. d: The OD_600_ value of bacterial solution after 5 h induction by pGEX-6p-3 and pGEX-6p-3-LpMYB4 under the treatment of 0.2 mM NaHCO_3_.

The results of protein induction are shown in Figure 4a. Under 1 mM IPTG induction conditions, the protein was induced for 0, 1, 3 and 5 h, respectively. The results showed that the protein appeared in a significantly thickened band at the position of 50 kDa after 1 h of induction at 1, 3 and 5 h of induction, and the expression reached the maximum at 5 h. The LpMYB4 was found to be abundantly present in the precipitates after a large number of induced (Figure 4a).

### Analysis of resistance to prokaryotic solution LpMYB4 protein expression under saline-alkaline stresses

There was no significant difference in the solution activity of pGEX-6p-3 strain and pGEX-6p-3-LpMYB4 strain after 5 h of induction when no stress treatment was performed (Figure 4ba); under 0.8 mM NaCl treatment, the OD_600_ values of pGEX-6p-3 and pGEX-6P-3-LpMYB4 solution were 0.906 and 1.253 (Figure 4bb); After 5 h under 0.1 M Na_2_CO_3_ treatment of pGEX-6p-3 and pGEX-6P-3-LpMYB4, the OD_600_ values of the bacterial liquid were 0.600 and 1.450, respectively (Figure 4bc); under 0.2 M NaHCO_3_ treatment, the OD_600_ values of the bacterial solution after 5 h of induction were 0.900 and 1.663 (Figure 4bd). Under the stress treatments of 0.1 M Na_2_CO_3_ and 0.2 M NaHCO_3_, the bacterial solution of pGEX-6P-3-LpMYB4 exhibited more pronounced saline and alkaline stress resistance compared to pGEX-6p-3. The growth of the bacterial solution was inhibited to different degrees under alkaline stress treatments, and the strain of pGEX-6P-3-LpMYB4 compared to the strain of pGEX-6p-3 showed stronger growth ability.

**Figure 5.**
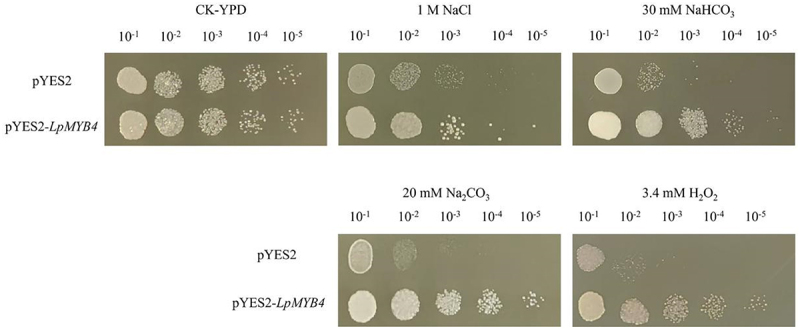
Resistance analysis of overexpression yeast under different abiotic stress resistance analysis of transgenic yeast under 1 M NaCl, 30 mM NaHCO_3_, 20 mM NaCO_3_ and 3.4 mM H_2_O_2_ stress growth of LpMYB4. Ten-fold dilutions of yeast cells containing pYES2 (upper line) and pYES2-LpMYB4 vector (lower line) were spotted on solid YPG media supplemented with the indicated stresses and grew at 30°C for 2 d. No treatment is a control (CK).

Under CK conditions, the growth of yeasts carrying *LpMYB4* and the control were essentially the same. Under the stress treatments of 1 M NaCl, 30 mM NaHCO_3_, 20 mM Na_2_CO_3_ and 3.4 mM H_2_O_2_ stress treatments, the growth of overexpressed yeast solution was significantly better than that of control (Figure 5).

### Resistance analysis of LpMYB4 overexpression tobacco under saline and alkaline stress

**Figure 6.**
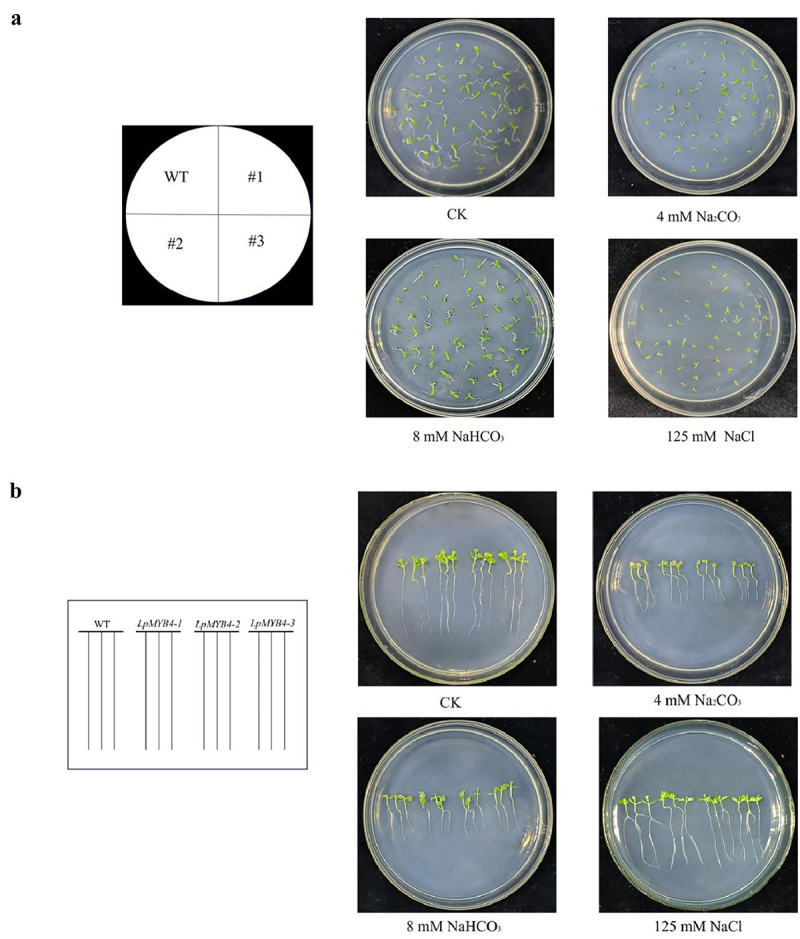
(a) Germination phenotypic analysis of wild-type tobaccos and LpMYB4 transgenic tobaccos under saline-alkaline stress. Tobacco seeds from the T3 generation, both wild-type and LpMYB4 transgenic, were taken and cultured for 7 d in 1/2 MS medium and 1/2 MS media supplemented with 4 mM Na_2_CO_3_, 8 mM NaHCO_3_, and 125 mM NaCl respectively. (b) Seeding growth of LpMYB4 transgenic plants under saline-alkaline stress. Wild-type tobacco seeds and T3 generation LpMYB4 transgenic plants were sown separately in the control group and groups in 1/2 MS medium supplemented with 125 mM NaCl, 8 mM NaHCO_3_ and 4 mM Na_2_CO_3_. The results were recorded after 14 d.

In order to investigate the effects of saline-alkaline stress on the germination of WT seeds and *LpMYB4* transgenic seeds, wild-type and *LpMYB4* transgenic T3 generation tobacco seeds were taken and cultured in 1/2 MS medium and 1/2 MS medium supplemented with 4 mM Na_2_CO_3_, 8 mM NaHCO_3_ and 125 mM NaCl, respectively, for 7 d. The observation results are shown in Figure 6a. All seeds germinated in 1/2 MS medium, and the seedlings were green. There was no significant difference between wild-type and *LpMYB4* transgenes visually. Under saline-alkaline stress of 4 mM Na_2_CO_3_, 8 mM NaHCO_3_, and 125 mM NaCl, the germination of wild-type seeds was significantly inhibited, while the young seedlings of germinated seeds were small and yellow. All the transgenic seeds germinated under saline-alkaline stress, and the seedlings were green and grew larger, which was better than the wild type. These results indicated that *LpMYB4* transgenic seeds had greater tolerance to saline-alkaline stress during germination.

In order to investigate the resistance analysis of transgenic plants seeding under saline-alkaline stress, wild-type tobacco seeding and T3 generation *LpMYB4* transgenic plants were arranged in 1/2 MS medium supplemented with 4 mM Na_2_CO_3,_ 8 mM NaHCO_3_ and 125 mM NaCl. The results obtained after 14 d are shown in Figure 6b. In the control medium, wild-type and transgenic seedlings presented green leaves, with long roots and uniform leaves, and there was no significant difference in growth potential between wild-type and transgenic seedlings. In 4 mM Na_2_CO_3_ medium, the leaves of the wild-type seedlings and transgenic seedlings developed less, and the leaves of wild-type leaves were completely yellow and transparent. In 8 mM NaHCO_3_ medium, both wild-type seedlings and transgenic seedlings were yellow, and the root length of the wild-type seedlings was still smaller than that of the wild-type seedlings. In 125 mM NaCl medium, wild-type and transgenic seedlings had the same growth condition of leaves, and the root length was smaller than that of transgenic seedlings. The results show that the *LpMYB4* gene affected seedlings under saline-alkaline stress and enhanced their resistance to saline-alkaline stress.

**Figure 7.**
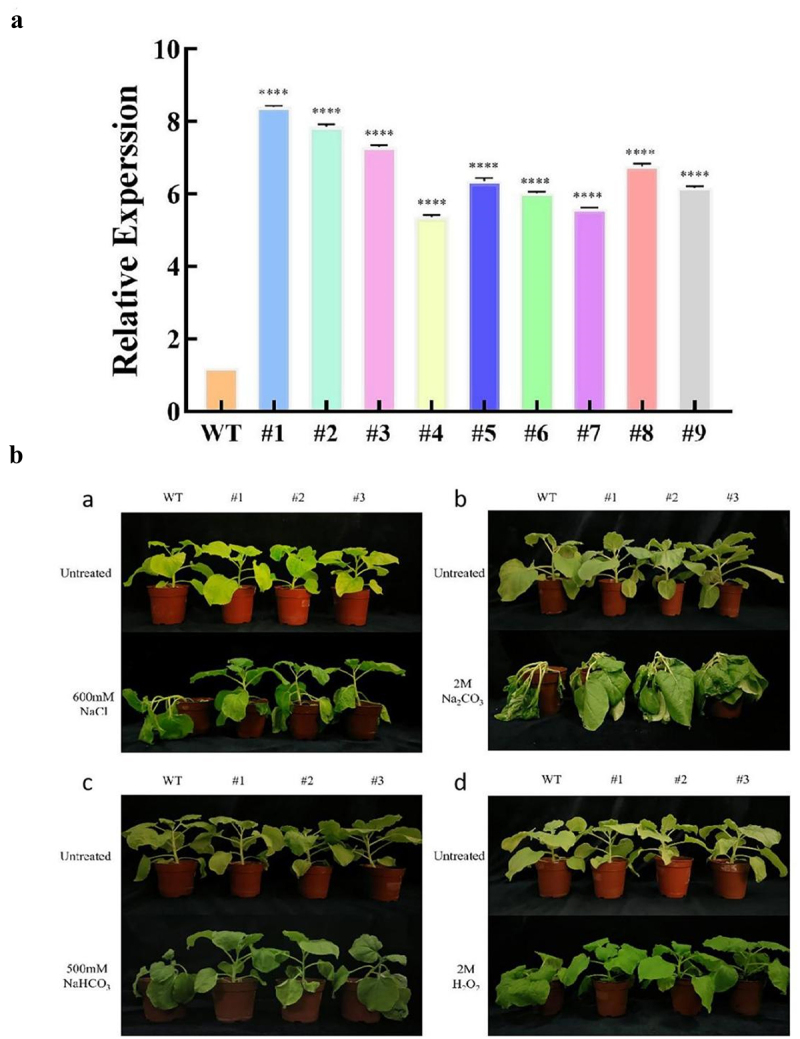
(a) Identification of overexpressed gene LpMYB4 tobacco. WT: wild type. #1-#10: LpMYB4 overexpression tobacco lines. (b) Phenotypic analysis of LpMYB4 transgenic plants under saline-alkaline stress. a: Plants were irrigated with 600 mM NaCl for 5 d. b: Plants were irrigated with 2 M Na_2_CO_3_ for 5 d. c: Plants were irrigated with 500 mM NaHCO_3_ for 5 d. d: Plants were irrigated with 2 M H_2_O_2_ for 5 d. ****respectively indicating that there is an extremely significant difference at p < 0.0001. Data represent mean ± SD with three replicates.

*LpMYB4* overexpression tobacco was identified by RT-PCR. The results in Figure 7a showed that the expression of *LpMYB4* was higher in overexpression lines than in wild-type lines, and overexpression lines #1, #2 and #3 were selected for subsequent experiments.

In the absence of saline stress, there was no significant difference in the phenotypes of overexpression and wild-type plants; when the plants were subjected to a series of stresses (600 mM NaCl, 2 M Na_2_CO_3_, 500 mM NaHCO_3_, and 2 M H_2_O_2_) for 5 d, the phenotypes of the overexpression plants and wild-type tobacco appeared to be significantly different, and the degree of wilting and collapsing of leaves in the overexpression plants was significantly lower than that

in the wild type. This suggests that the *LpMYB4* confers superior longevity and growth capacity under saline stress, *LpMYB4* helps to enhance the adaptability as well as resistance of plants in saline soils (Figure 7b). Under a series of treatments (600 mM NaCl, 2 M Na_2_CO_3_, 500 mM NaHCO_3_, and 2 M H_2_O_2_), the chlorophyll content of overexpressed tobacco was significantly higher than that of the wild type. The results suggest that stresses may affect plant growth by influencing chlorophyll content, and the chlorophyll contents of overexpressed plants were all significantly higher than those of the wild type under saline stress treatments, representing that they were subjected to less saline injury (Figure S2).

As one of the most stable forms of reactive oxygen species in plants, excessive accumulation of hydrogen peroxide can cause damage to the plant cell membrane system and impair intracellular metabolic processes. The results showed that the *LpMYB4* gene plays a role in scavenging hydrogen peroxide in plants, and the hydrogen peroxide content of overexpressed *LpMYB4* lines was significantly lower than that of the control group under saline treatment, i.e., under saline and alkaline stress, the overexpressed plants had lower levels of hydrogen peroxide in the body and less cellular damage (Figure S3a). Superoxide anion is the precursor of all reactive oxygen radicals in plants, and excessive accumulation of superoxide anion can cause serious adverse effects on plant growth. The results showed that the *LpMYB4* gene could scavenge superoxide anion in plants; the accumulation of superoxide anion in overexpression plants was significantly lower than that in the wild type under saline and alkaline stress, and the cell damage was less (Figure S3B). The relative conductance of overexpressed tobacco was significantly lower than that of the wild type under a series of saline stress treatments (Figure S3C), suggesting that the gene *LpMYB4* reduces saline-alkaline damage to plant cell membranes. Under saline and alkaline stress, reactive oxygen species (ROS) accumulate in large quantities in plants, thus causing damage to plant cells. The ROS content in overexpressed tobacco is significantly lower than that in the control group; in order to investigate the mechanism and reasons for the changes in ROS in plants, we measured the activities of antioxidant enzymes in plants. POD and SOD are important scavengers of peroxides and free radicals in plants and play an important protective role during the process of plant cell damage. The results showed that the activities of POD and SOD in overexpressed tobacco were significantly higher than those of the wild type under saline and alkaline stress treatments (2 M H_2_O_2_, 600 mM NaCl, 2 M Na_2_CO_3_, 500 mM NaHCO_3_) (Figure S3D&E). After saline stress treatment, the antioxidant enzyme activities of the control group were significantly lower than those of the overexpression lines, which indicated that the LpMYB4 gene could help plants to scavenge reactive oxygen species, thus preventing the damage of ROS to the plant cells, and enhancing the plant’s ability to survive under saline stress.

### Screening for LpMYB4 interacting proteins

Eleven proteins were successfully compared to these colonies by BLAST in NCBI. Six of them have been shown to be associated with plant saline-alkali tolerance (Table S2).

### Bimolecular luciferase complementation assay

**Figure 8.**
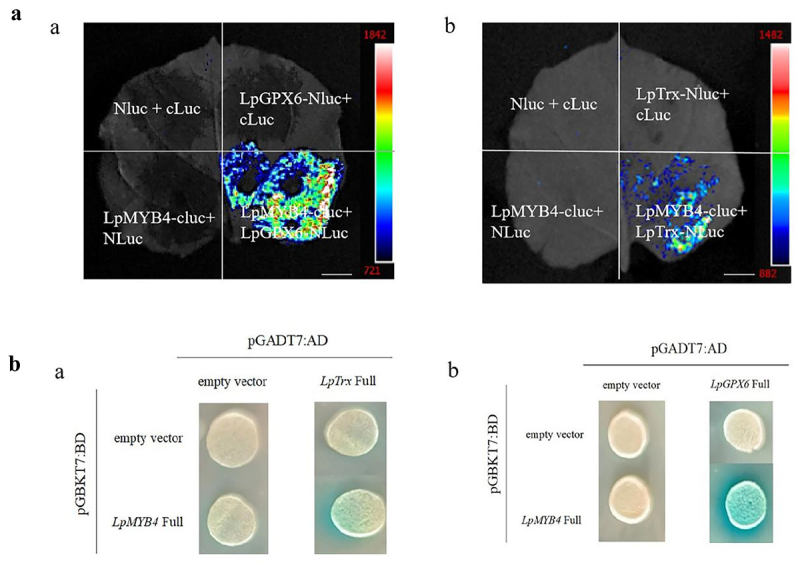
A: Complementary assay of LpMYB4 and LpGPX6 or LpMYB4 and LpTrx bimolecular luciferase. a: Complementary assay of LpMYB4 and LpGPX6 bimolecular luciferase. b: Complementary assay of LpMYB4 and LpTrx bimolecular luciferase. In the first quadrant: both NLuc and cLuc were unloaded, the second quadrant: cLuc was unloaded, the third quadrant: NLuc was unloaded and the fourth quadrant: LpMYB4-CLuc andLpgpx6-NLuc. Scale bar = 1 cm. b: The yeast two-hybrid results are shown in Figure. a: pGBKT7-LpMYB4 and pGADT7-LpTrx co-conversion validation. On SD-Trp-Leu-His plus X-gal media, the strains cotransformed with pGBKT7-LpMYB4 and pGADT7-LpTrx were able to establish blue colonies, whereas the control group was unable to do that. b: pGBKT7-LpMYB4 and pGADT7-LpGPX6 co-conversion validation. On SD-Trp-Leu-His plus X-gal media, the strains cotransformed with pGBKT7-LpMYB4 and pGADT7-LpGPX6 were able to establish blue colonies, whereas the control group was unable to do that.

MYB acts as an antioxidant to scavenge reactive oxygen species (ROS) to combat adversity, GPX6 catalyzes the reduction of peroxides to protect cells from oxidative damage, and Trx enhances plant resistance to salinity stress by maintaining osmotic homeostasis and scavenging reactive oxygen species (ROS). We speculated whether MYB and GPX6, MYB and Trx could bind and interact with each other to jointly reduce the effects of adversity on plants. Using the bimolecular luciferase complementation assay, it was found that LpMYB4 and LpTrx can interact with each other, and LpMYB4 and LpGPX6 can also interact (Figure 8a).

### Yeast two-hybrid experiment

For further verification, pGADT7-LpTrx and pGADT7-LpGPX6 was successfully constructed. The strains cotransformed with pGBKT7-LpMYB4 and pGADT7-LpTrx or pGBKT7-LpMYB4 and pGADT7-LpGPX6 could grow blue colonies on SD-Trp-Leu-His plus X-α-gal medium, while there were no blue colonies growth in the control group (Figure 8b). These results indicate that LpMYB4 and LpTrx proteins can interact with each other.

## Discussion

Abiotic stresses such as saline-alkaline stress seriously affect the growth and survival of plants. *L. pumilum* can grow well in drought, saline, cold and poor soil nutrition. The transcription factor MYB is involved in plant saline-alkaline stress resistance through the regulation of downstream target genes.^18,19^

We cloned *LpMYB4* gene from *L. pumilum*, The MYB4 belongs to subgroup 4-MYB subprotein family.^6,7^ LpMYB4 contained MYB-DNAbinding structural domains, PLN03091, REB1, SANT, and other typical structural domains of MYB, performed preliminary bioinformatics analysis and phylogenetic tree construction, and found that the evolutionary relationship was close to the bamboo and maize *MYB4* genes. The *MYB* gene structure in maize and Arabidopsis were highly conserved, indicating that they were originally compact in size., Subgroup-specific conserved motifs outside the MYB domain may reflect functional conservation,^20^ this meaning that *LpMYB4* may have similar function with *AtMYB4*. *AtMYB4enhanced protection against oxidative damage*,^21^ and plays dual roles in flavonoid biosynthesis^6^ So what functions does LpMYB4 have? At present, there are few studies on the molecular mechanism of *MYB4* gene involved in *L. pumilum* stress, and we mainly explore the mechanism of salinity tolerance of *MYB4* gene.

RT-qPCR identified the highest *LpMYB4* expression in the bulb, followed by flower, leaf, seed, and root; *MYB4* gene could be induced by saline-alkaline stress, and the transcription level reached a peak after 100 mM NaCl induction for 1 h, and then decreased.^7^ *MdMYB4* expression initially increased in response to NaCl, but then decreased at NaCl concentrations that exceeded 150 mM.^22^ *MYB4* response to NaCl, but few researches for *MYB4* response to NaHCO3, In this study, *LpMYB4* expression level reached a peak after stress induction for 6 h, different stresses maybe induced gene expression at different times.

*MdMYB4* was mainly expressed in the nucleus of onion epidermal cells (MdMYB4 enhances apple callus salt tolerance by increasing MdNHX1 expression levels), subcellular localization identified LpMYB4 protein in the nucleus, consistent with its identity as a transcription factor.

Resistance experiments were performed in prokaryotes and eukaryotes. *E. coli* or yeast containing recombinant *LpMYB4* grew significantly better than the wild-type control strain under saline-alkaline treatment. Suggests that *LpMYB4* may be involved in the stress response in *E. coli*, making the transgenic *E. coli* more salt-tolerant.

The sensitivity of the mutant to NaCl was significantly increased, and the tolerance of overexpressed plants to saline-alkaline stress was significantly enhanced.^7^

Overexpression of *MYB4* can improve the drought resistance of rice and apple plants,^16^ Moreover, the *MYB4* is also related to the synthesis of lignin and anthocyanin and may be one of the mechanisms to enhance plant abiotic stress tolerance.^13,14^ After receiving a series of salinity stress, the germination and seedling growth of overexpressed plants were better than those of wild type, wilting and lodging of overexpressed *LpMYB4* tobacco lines were significantly lower than WT. Under saline-alkaline stress, the chlorophyll content of overexpressed *LpMYB4* tobacco was significantly higher than that of wild type; the relative conductivity of overexpressed tobacco was significantly lower than that of wild type, which indicates that *LpMYB4* can reduce the damage to the plant cell membrane;

Transgenic *BpMYB4* plants have higher proline and lower MDA content. In addition, they found that overexpression of *BpMYB4* can clear H_2_O_2_ and O_2_^−^ .^10^ In this study, the activity of antioxidant enzymes (POD,SOD) in the overexpressed *LpMYB4* line was significantly higher than that in the control group, which helped to clear the reactive oxygen species in the body. This showed that *LpMYB4* can clear ROS.

Trx can directly participate in the scavenging of ROS as a reducing agent for these proteins, we selected two proteins related to H_2_O_2_ clearance for Luc experiments to test target proteins that interact with LpMYB4.

Proteins in an organism need to interact with other proteins to function, In Arabidopsis, AtMYB4 was implicated in interaction with bHLH proteins and a WD40 repeat protein,^23^ MdMYB4 can bind to the *MdNHX1* promoter. MYB4 directly represses the transcription of the *C4H* (*cinnamate-4-hydroxylase*) gene.^19^ As a key transcription factor in plants, MYB4 may activate the expression of these genes by binding to the promoters of target genes (HKT1, NHX4, SOS1 and SOS3) to improve the tolerance of plants to saline-alkali stress.^7^

Eight positive interacting proteins were obtained using yeast two hybrids, from these Selected interacting proteins, Thioredoxin (Trx) and Glutathione peroxidases (GPXs) participated in the plant ROS response process, The Trx can directly participate in the scavenging of ROS as a reducing agent.^24,25^

**Figure 9.**
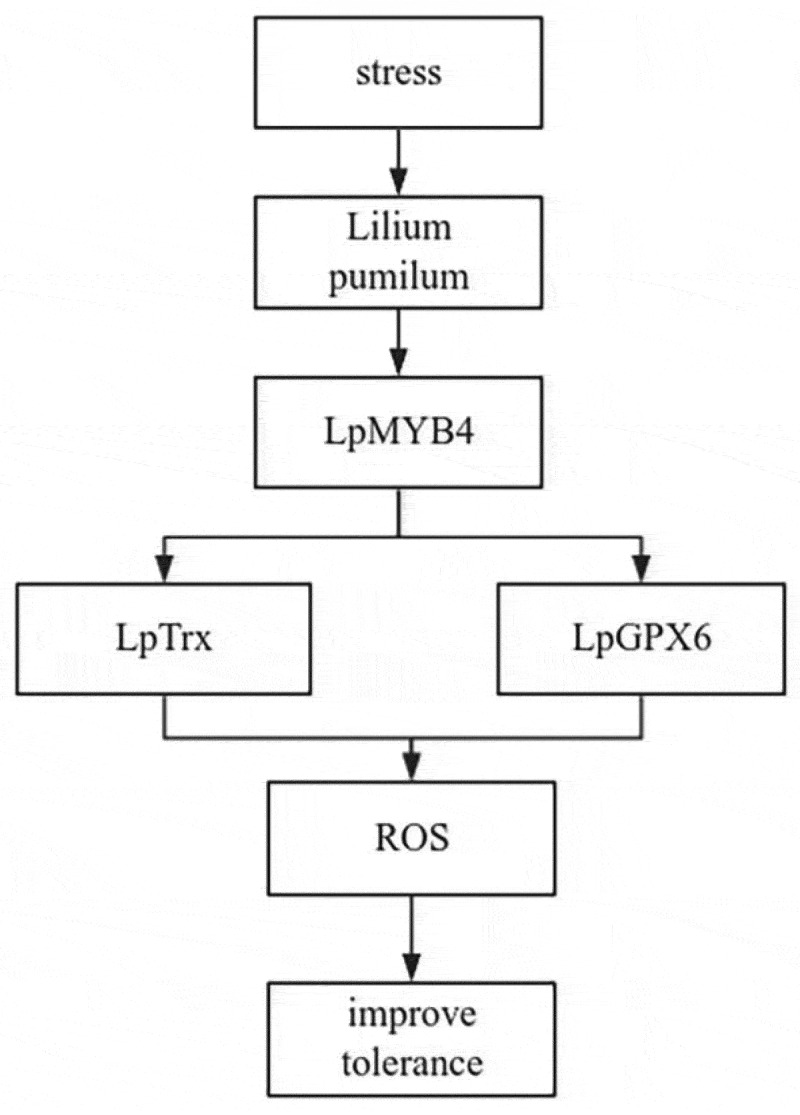
Model of regulatory mechanisms of the gene LpMYB4 in response to saline-alkali tolerance. LpMYB4 interacts with LpTrx, LpGPX6 scavenged ROS, resulting in improved tolerance to saline-alkaline stress.

The AtGPX6 (glutathione peroxidases) gene was reported to be much expressed in most tissues during plant development and the gene transcription level was evidently increased under several abiotic stress.^25,26^ Glutathione peroxidases (GPXs) play important roles in the removal of peroxides and the protection of cells from peroxides through catalyzing the reduction of hydrogen peroxide (H_2_O_2_) or organic peroxides into water or corresponding alcohol.^27,28^ AtGPX6 exhibits crucial functions in ROS homeostasis as well as the response to stress signals.^29^ In this study, the firefly luciferase complementation imaging assay and two were performed to preliminarily prove the protein-protein interaction relationship,

From these results, we present a model for explaining how *LpMYB4* responds to stresses. *LpMYB4* mediates ROS homeostasis, *LpMYB4* expression improved under the stresses, LpMYB4 interaction with LpTrx or LpGPX6, LpTrx or Lpgpx6 regulating ROS homeostasis, so LpMYB4 participate in the regulation of stresses through mediating ROS homeostasis by interacting with LpTrx or LpGPX6 (Figure 9).

## Conclusions

In this study, we cloned the *LpMYB4* gene from the *L. liumpum* and conducted several bioinformatics analyses. RT-qPCR confirmed that the *LpMYB4* was most highly expressed in the bulbs of *L.liumpum*. Subcellular localization experiments showed that the LpMYB4 localized mainly in the nucleus, which was in line with our expectations. Constructing prokaryotic and eukaryotic expression strains of the *LpMYB4* and conducting bacterial solution resistance experiments revealed that transgenic *E. coli* and yeast showed better growth than the wild-type strains under saline and alkaline stress conditions. The salinity resistance analysis of *LpMYB4* overexpression tobacco revealed that it had significant growth advantages under saline stress conditions, with lower leaf wilting and collapsing than wild-type. Measured physiological indexes also showed that the *LpMYB4* gene could enhance plant tolerance. Screening for *LpMYB4* interacting proteins identified 11 potential candidates; out of which six proteins were related to enhancing plants’ abiotic stress tolerance.

## Supplementary Material

Supplementary_file_docx.docx
