# Supplementary material for: MYB4 in Lilium pumilum affects plant saline-alkaline tolerance
Source: Plant Signal Behav. 2024 Jul 14;19(1):2370724. doi: 10.1080/15592324.2024.2370724 (PMC11249031; doi:10.1080/15592324.2024.2370724)
Supplement: Supplementary_file_docx.docx [file KPSB_A_2370724_SM1505.docx]

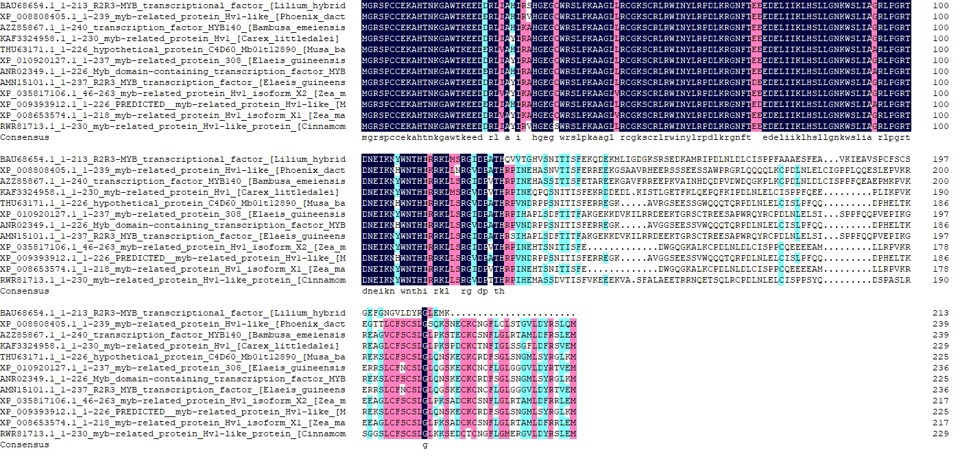


Fig. S1 Homologous sequence comparison of protein LpMYB4. Homology levels of 100% are indicated in black, ≥ 75% in pink, and ≥ 50% in cyan in the graph.


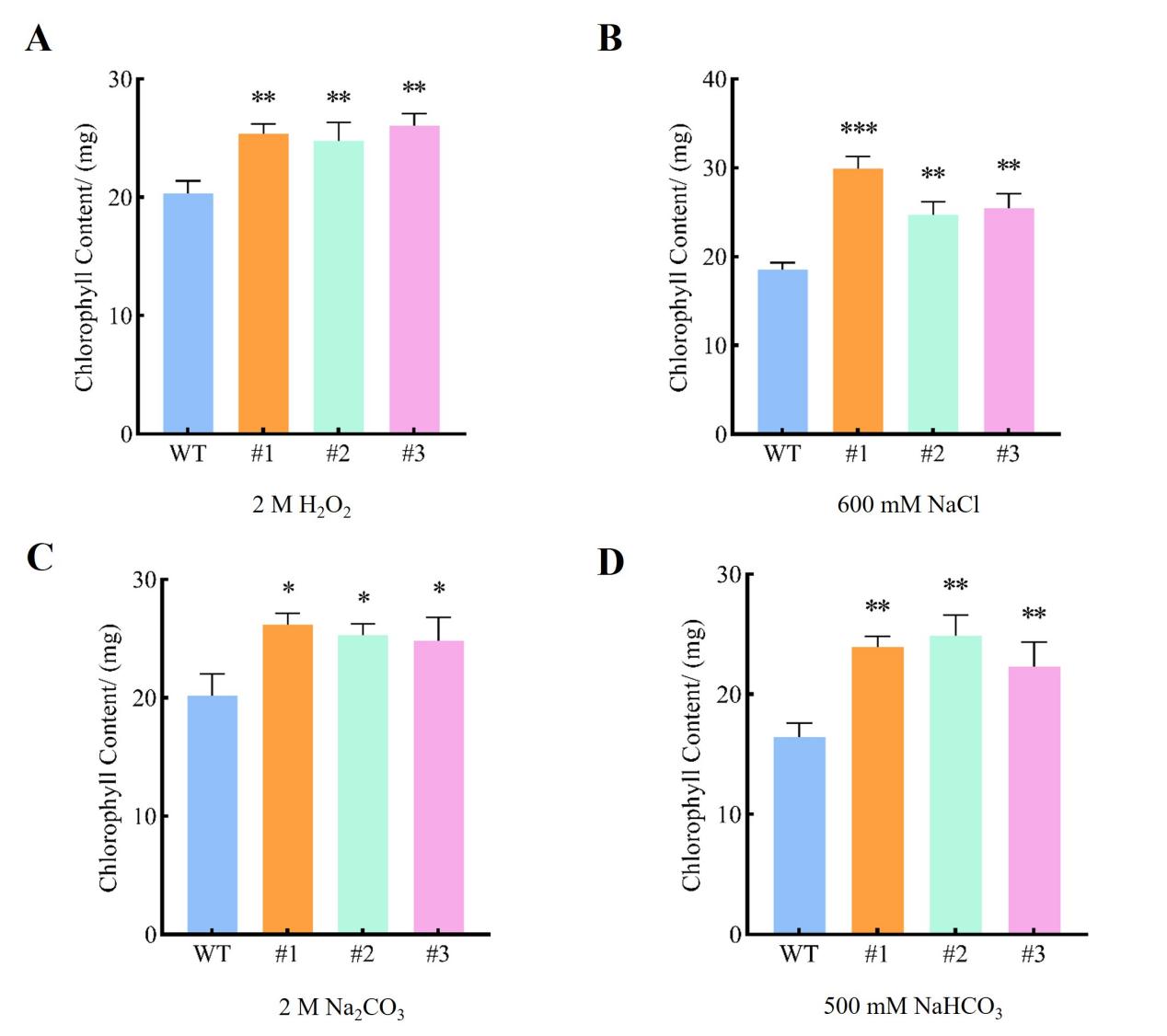


Fig. S2 Determination of chlorophyll content in LpMYB4 tobacco under saline-alkaline stress. SPAD chlorophyll meter was used to measure the chlorophyll contents of tobacco under different saline-alkaline stress.

A: Chlorophyll content of LpMYB4 tobacco under 2 M H_2_O_2_ stress.

B: Chlorophyll content of LpMYB4 tobacco under 600 mM NaCl stress.

C: Chlorophyll content of LpMYB4 tobacco under 2 M Na_2_CO_3_ stress.

D: Chlorophyll content of LpMYB4 tobacco under 500 mM NaHCO_3_ stress.

*,**, and*** respectively indicate that there is an extremely significant difference at P < 0.05, P < 0.01 and P<0.0001. Data represent mean ± SD with three replicates.


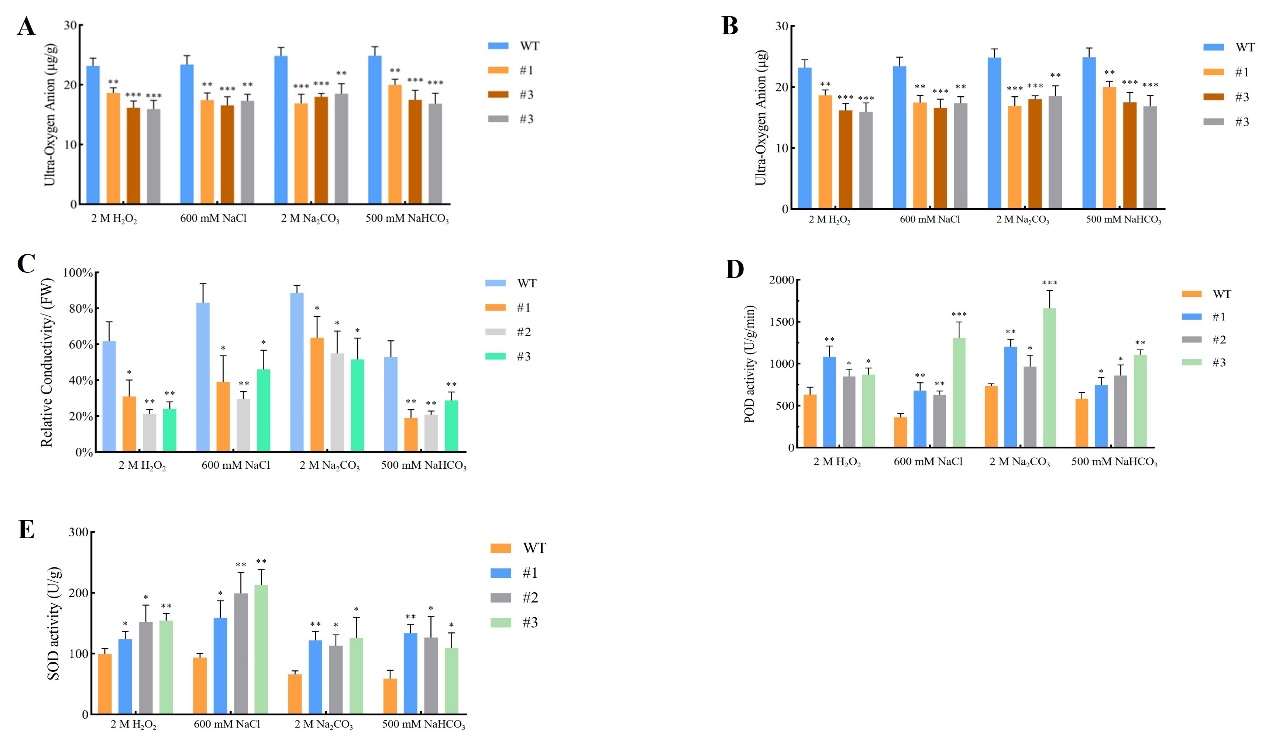


Fig. S3 Physiological index parameters of *LpMYB4* tobacco under saline stress. Analysis of physiological indexes of tobacco plants under saline stress."*" indicates a significant difference and "**" indicates a highly significant difference. A: Hydrogen peroxide content of tobacco under salinity stress. (2 M H_2_O_2_, 600 mM NaCl, 2 M Na_2_CO_3_, 500 mM NaHCO_3_). B: Superoxide anion content of tobacco under saline stress. (2 M H_2_O_2_, 600 mM NaCl, 2 M Na_2_CO_3_, 500 mM NaHCO_3_). C: Relative conductivity of tobacco under saline stress. (2 M H_2_O_2_, 600 mM NaCl, 2 M Na_2_CO_3_, 500 mM NaHCO_3_). D: POD activity of tobacco under saline stress (2 M H_2_O_2_, 600 mM NaCl, 2 M Na_2_CO_3_, 500 mM NaHCO_3_). E: SOD activity of tobacco under saline stress (2 M H_2_O_2_, 600 mM NaCl, 2 M Na_2_CO_3_, 500 mM NaHCO_3_). *, **and*** respectively indicate that there is an extremely significant difference at P < 0.05, P < 0.01 and P<0.0001. Data represent mean ± SD with three replicates.

**Table S1. Primers used in this study**

| Primer name | primer sequence |
| --- | --- |
| *LpActin F* | GCATCACACCTTCTACAACG |
| *LpActin R* | GAAGAGCATAACCCTCATAGA |
| *RT-qPCR LpMYB4 F* | AGAAGACGACCGGCTTATCG |
| *RT-qPCR LpMYB4 R* | CCGGTCACAGCTTGATGAGT |
| *LpMYB4- Bam*HⅠ *F* | GGATCCATGGGGAGGTCTCCAT |
| *LpMYB4-Xhol*Ⅰ *R* | CTCGAGTCATTTCATCTCAAGGCCT |
| *LpMYB4-Xba*Ⅰ *R* | TCTAGATTTCATCTCAAGGCCTCTATAG |
| *LpMYB4-Eco*RI *F* | GAATTCATGGGGAGGTCTCCAT |
| *LpMYB4-Bam*HI *R* | GGATCCTCATTTCATCTCAAGGC |
| *LpMYB4-sal*I *R* | GTCGACATTTCATCTCAAGGCC |
| *LpMYB4-Xba*Ⅰ *F* | TCTAGAATGGGGAGGTCTCCAT |
| T7 *-F* | TAATACGACTCACTATAGGGC |
| 3*-AD R* | AGATGGTGCACGATGCACAG |
| *LpGPX6*-SalI R | GTCGACGAGCAGTTTCTTGATA |
| *LpGPX6*-KpnI F | GGTACCATGCTCCGTCCTCATC |

**Table S2. LpMYB4 interacting proteins**

| Protein Name | Function (related to abiotic plant stresses) |
| --- | --- |
| Genome assembly, chromosome: 5 | No relevant features found |
| BAX inhibitor 1 (BI1) | Improves plant tolerance to heat, drought and salinity; inhibits stress-induced cell death and improves plant salinity tolerance |
| Nascent polypeptide-associated complex (NAC), alpha subunit family protein | Increases plant chlorophyll and proline content, improves ionic homeostasis, and increases drought, cold, and salinity tolerance. |
| GDP-binding protein | Increase in alkaline phosphatase (ALP) activity and tolerance to drought and saline stress in plants |
| Uncharacterized Plant/protein mRNA | No relevant features found |
| Uncharacterized protein (AT1G34010) | No relevant features found |
| P-loop nucleoside triphosphate hydrolase superfamily protein | Inhibited plant antioxidant enzyme activities, reduced ROS levels, and increased plant tolerance to salt stress |
| Glutathione peroxidase 6 Gene-GPX6 | Catalyses the reduction of hydrogen peroxide, organic hydroperoxides and lipid hydroperoxides, thereby protecting cells from oxidative damage |
| GDSL-like Lipase/Acyl hydrolase superfamily protein | Transcriptome analysis showed that its expression was up-regulated under salinity stress treatment, which was hypothesized to be associated with the enhancement of plant resistance to salinity stress |
| Ecotype Col-0 mitochondrion | No relevant features found |
| Mesoderm induction early response protein (ATI1) | No relevant features found |
| Thioredoxin superfamily protein (HCF164) | Enhancement of plant resistance to saline and alkaline stress through maintenance of osmotic homeostasis, scavenging of reactive oxygen species (ROS), and modulation of plant stress response |
